# Focal Salvage Treatment of Radiorecurrent Prostate Cancer: A Narrative Review of Current Strategies and Future Perspectives

**DOI:** 10.3390/cancers10120480

**Published:** 2018-12-03

**Authors:** Marieke van Son, Max Peters, Marinus Moerland, Linda Kerkmeijer, Jan Lagendijk, Jochem van der Voort van Zyp

**Affiliations:** Department of Radiotherapy, University Medical Center Utrecht, Heidelberglaan 100, 3584 CX Utrecht, The Netherlands; M.Peters-10@umcutrecht.nl (M.P.); M.A.Moerland@umcutrecht.nl (M.M.); L.Kerkmeijer@umcutrecht.nl (L.K.); J.J.W.Lagendijk@umcutrecht.nl (J.L.); J.R.N.vanderVoortvanZyp@umcutrecht.nl (J.v.d.V.v.Z.)

**Keywords:** prostate cancer, localized recurrence, focal therapy, salvage

## Abstract

Over the last decades, primary prostate cancer radiotherapy saw improving developments, such as more conformal dose administration and hypofractionated treatment regimens. Still, prostate cancer recurrences after whole-gland radiotherapy remain common, especially in patients with intermediate- to high-risk disease. The vast majority of these patients are treated palliatively with androgen deprivation therapy (ADT), which exposes them to harmful side-effects and is only effective for a limited amount of time. For patients with a localized recurrent tumor and no signs of metastatic disease, local treatment with curative intent seems more rational. However, whole-gland salvage treatments such as salvage radiotherapy or salvage prostatectomy are associated with significant toxicity and are, therefore, uncommonly performed. Treatments that are solely aimed at the recurrent tumor itself, thereby better sparing the surrounding organs at risk, potentially provide a safer salvage treatment option in terms of toxicity. To achieve such tumor-targeted treatment, imaging developments have made it possible to better exclude metastatic disease and accurately discriminate the tumor. Currently, focal salvage treatment is being performed with different modalities, including brachytherapy, cryotherapy, high-intensity focused ultrasound (HIFU), and stereotactic body radiation therapy (SBRT). Oncologic outcomes seem comparable to whole-gland salvage series, but with much lower toxicity rates. In terms of oncologic control, these results will improve further with better understanding of patient selection. Other developments, such as high-field diagnostic MRI and live adaptive MRI-guided radiotherapy, will further improve precision of the treatment.

## 1. Introduction

Prostate cancer is the most diagnosed male cancer in developed countries. Frequently diagnosed at an early stage, with opportunistic prostate-specific antigen (PSA) screening increasing the incidence, the search for optimal and patient-tailored treatment is of growing significance. In the setting of localized recurrent prostate cancer after primary whole-gland radiotherapy, the standard of care now consists of palliative androgen deprivation therapy (ADT). This only has a temporary suppressive effect and is associated with harmful side-effects. On the other hand, treatments with curative intent such as salvage prostatectomy or whole-gland radiotherapy also convey serious toxicity risks and should only be offered to highly selected patients [1]. This leaves a gap in the treatment arsenal for radiorecurrent prostate cancer. Here, focal ablative treatment might meet the need; with lower toxicity risks, it could postpone palliative hormonal treatment or perhaps even avoid it altogether. Within this narrative review, an overview is provided of the developments in primary prostate cancer care, current strategies on how to deal with localized prostate cancer recurrences, and future perspectives with respect to focal salvage treatment.

## 2. Whole-Gland Primary Radiotherapy

For whole-gland treatment of intermediate- to high-risk prostate cancer in the primary setting, radiotherapy has evolved as a suitable modality. It is comparable to prostatectomy in terms of cancer control, while both are associated with their respective side-effects [2]. Several developments over the last decades increased the use of radiotherapy for the primary treatment of prostate cancer. Intensity-modulated radiation therapy (IMRT) and volumetric modulated arc therapy (VMAT) are increasingly adopted as external beam radiation therapy (EBRT) techniques, using fiducial gold markers for position verification. Both are able to substantially reduce the dose to surrounding organs at risk (in particular rectum and bladder) due to a more conformal dose distribution [3,4]. Although radiation therapy traditionally entailed a lengthy treatment with smaller daily fractions over 6–7 weeks, hypofractionation seems to provide comparable tumor control, against acceptable toxicity profiles [5,6,7,8,9]. The rationale behind using higher doses in fewer fractions comes from data describing a lower α/β-ratio of prostate cancer than previously thought. Despite ambiguous recommendations from different large trials, hypofractionated radiotherapy is increasingly adopted in guidelines worldwide [10].

While external beam techniques are generally delivered fractionated, internal radiation using brachytherapy is increasingly performed in a single procedure. Originally, low-dose-rate brachytherapy (using iodine-125 seeds) was mainly used for low- to intermediate-risk patients. Currently, there is an increase in the treatment of higher-risk disease with high-dose-rate brachytherapy, providing comparable cancer control rates to other primary treatments [11,12,13]. As compared to iodine-125 seeds, high-dose-rate brachytherapy offers the advantage of higher dose control via the approach of adjusting source dwell times and positions. The steep dose decline of brachytherapy makes it possible to further escalate the dose to the tumor, without compromising the dose constraints for the organs at risk [13]. This feature can also be used to deliver a concurrent tumor boost next to whole-gland EBRT techniques, thereby further increasing the therapeutic efficacy for intermediate- to high-risk disease [14].

## 3. Recurrence Risk and Location

Although dose escalation is increasingly adopted, recurrent prostate cancer after primary radiotherapy remains common. A recent series of 2694 patients treated with doses above 78 Gy revealed 10-year biochemical recurrence risks of approximately 10%, 23%, and 44% in low-, intermediate-, and high-risk patients, respectively [15]. Biochemical recurrences according to the Phoenix definition (i.e., PSA nadir + 2.0 ng/mL) preceded the development of distant metastases and death due to prostate cancer by 5.4 years and 10.5 years, respectively. In patients with a reasonable life expectancy, management of these recurrences is, therefore, often necessary to prevent cancer-related complications and mortality.

Primary prostate cancer is often a multifocal process [16,17], with a hypothesized “index lesion” driving metastatic potential [18,19]. Within this hypothesis, it is thought that synchronous lesions outside the index lesion are secondary insignificant cancers which lie dormant [20]. After primary whole-gland radiotherapy, several series showed that recurrences nearly all (89–100%) regrow at the site of the primarily largest and/or highest-grade index lesion [21,22,23,24,25]. This indicates that the malignant remnant causes biochemical failure, while secondary indolent tumor foci are successfully treated by the primary radiation course. Building on this, the rationale behind focal treatment in the localized radiorecurrent setting becomes clear. Although the index lesion hypothesis remains controversial due to a lack of robust evidence, long-term oncological efficacy data of focal salvage treatments in the future might help to either support or undermine this view.

## 4. Traditional Approach to Radiorecurrent Prostate Cancer

The treatment of prostate-confined recurrences after primary radiotherapy is called salvage and will be denoted as such in the subsequent part of this review. Within the literature, there are reasonably large series available describing the results of salvage treatments directed at the entire prostatic volume. These series include salvage radical prostatectomy (SRP) [26], whole-gland salvage cryotherapy [27,28], whole-gland salvage high-intensity focused ultrasound (HIFU) [29,30], and, in increasingly larger series, whole-gland salvage brachytherapy [31,32,33]. These studies show an approximate five-year biochemical failure-free survival (bFFS) of 50–60%, thereby postponing the use of palliative ADT with its associated toxicity [34]. However, due to previous radiation damage to organs at risk, toxicity of secondary surgery or radiation can be deleterious. Severe genitourinary (GU) and gastrointestinal (GI) toxicity, requiring operative intervention to resolve, are observed in about 30% of patients, with erectile dysfunction (ED) often presents in 100% of cases post-salvage [35]. For this reason, whole-gland techniques remain unpopular amongst treating physicians, with only 2% of patients receiving any form of salvage curative treatment. The other 98% receive ADT, either immediately or deferred [36]. These patterns are also observed in large national databases, such as the Cancer of the Prostate Strategic Urological Research Endeavor (CaPSURE) database from the United States (US) [37].

## 5. Focal Treatment of Radiorecurrent Prostate Cancer

With recurrences often being localized and unifocal (mainly at the “index lesion” site), a salvage treatment directed solely at the recurrent tumor lesion seems rational. Especially considering the narrow therapeutic ratio (treatment efficacy versus treatment-related toxicity) in the recurrent setting, focal treatment provides a promising alternative: a second chance at achieving local control, with minimal burden to the patient in terms of side-effects.

### 5.1. Diagnostic Assessment

#### 5.1.1. Excluding Metastatic Disease

The success of focal salvage treatment starts with adequate exclusion of metastatic disease. More dated series of whole-gland salvage treatments often show substantial failure rates due to inadequate pre-treatment diagnosis of metastases. For example, technetium-99m bone scintigraphy was often used to exclude bone metastases, which only achieves acceptable diagnostic accuracy in patients with higher-risk disease characteristics (PSA >20, Gleason ≥8) [38]. Furthermore, studies regarding computed tomography (CT) and/or magnetic resonance imaging (MRI) for nodal disease staging demonstrated poor diagnostic accuracy [39], since lymph node diameter and morphology are inadequate predictors for nodal invasion. Positron-emission computed tomography (PET/CT), however, is recommended as the standard diagnostic modality to assess metastatic disease in the recurrent setting. It offers the advantage of concurrently evaluating bony and nodal metastatic disease. Different PET tracers are used, with choline and fluoride as the most abundant originally [40,41,42]. Negative predictive values of up to 100% were reported, although the range observed in the reported literature is substantial. Thus far, the most promising PET technique seems to be ^68^Ga prostate-specific membrane antigen (PSMA)-PET/CT, with a radiotracer binding more specifically to a cellular protein overexpressed on 95% of prostate cancer cell membranes. High diagnostic accuracy is attained for both intra-prostatic lesions, as well as lymph node and bone metastases, even at low PSA values (<2 ng/mL) [43,44]. Available since 2013 [45], PSMA-PET/CT quickly became a routine form of targeted molecular imaging in countries across Asia, Australia, and Europe [46]. Currently, diffusion-weighted whole-body MRI is also being investigated for assessment of bone metastases in the recurrent setting, although PET/CT seems superior [47,48].

#### 5.1.2. Assessing and Targeting Intra-Prostatic Disease

After exclusion of metastatic disease, assessment of intra-prostatic disease is necessary to adequately target the recurrent lesion. In the past, salvage treatments had to be aimed at the whole prostate gland since localization of the recurrent nodule was inadequate. Currently, this is possible with the use of multi-parametric MRI (mp-MRI), offering both morphological and functional information with T2-weighted, dynamic contrast-enhanced (DCE), and diffusion-weighted imaging (DWI). In the primary setting, the diagnostic accuracy of mp-MRI for the detection of clinically significant intra-prostatic disease seems adequate with a sensitivity of 93% [49,50]. Although smaller (secondary) tumor foci are still occasionally missed (even when harboring higher-grade cancers), mp-MRI is often able to detect the larger index tumor [51]. Because of the relatively high contrast of fibrotic prostatic tissue with viable tumor tissue in a previously irradiated prostate, DCE- and DWI-MRI are especially capable of adequately detecting radiorecurrent lesions [52,53,54].

However, in the setting of treatment failure evaluation, the interpretation of mp-MRI is often complicated by treatment-related anatomic and functional changes. Radiologists should be familiar with the findings that are associated with the type of treatment the patient previously received. For instance, T2 hypo-intense intraprostatic lesions can be difficult to distinguish within a diffusely hypo-intense prostate caused by previous irradiation. Although there are no established guidelines for characterizing possible local tumor relapses on mp-MRI, there is an increasing amount of literature discussing the differences between normal post-treatment patterns and suspicious recurrence findings [55,56,57,58,59].

**Figure 1:**
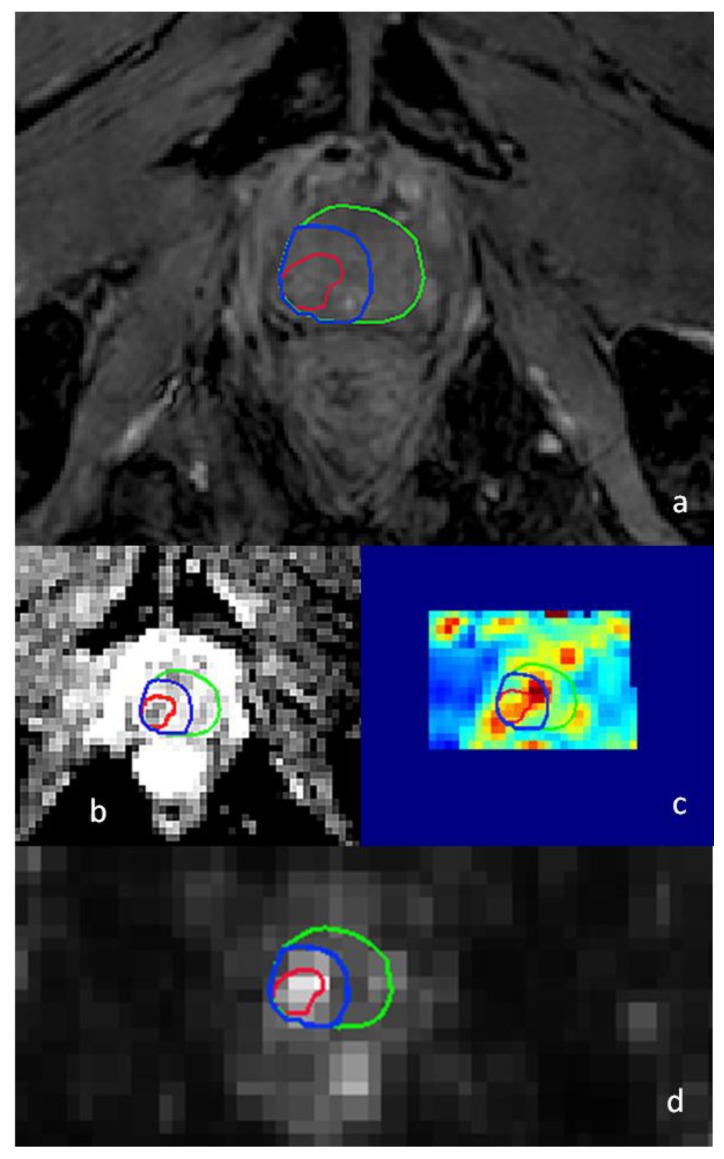
Recurrent prostate cancer lesion on diagnostic 3-T multiparametric magnetic resonance imaging (mp-MRI) (**a**–**c**) and prostate-specific membrane antigen positron-emission computed tomography (PSMA-PET/CT) (**d**). The suspect lesion is visible in the right peripheral zone of the apex. Delineations of the prostate (green), gross tumor volume (GTV, red), and clinical target volume (CTV, blue) are displayed. (**a**) T2-weighted MRI; (**b**) apparent diffusion coefficient (ADC) map of diffusion-weighted imaging (DWI)-MRI; (**c**) K-trans map of dynamic contrast-enhanced (DCE)-MRI; (**d**) ^68^Ga-PSMA-PET/CT.

The combination of ^68^Ga-PSMA-PET/CT with mp-MRI could provide an even higher accuracy in detecting and delineating intra-prostatic disease [60] (see Figure 1 for example). A retrospective analysis on the diagnostic value of ^68^Ga-PSMA-PET/CT in the recurrent setting revealed a negative predictive value (NPV) and positive predictive value (PPV) of 91.4% and 100%, detecting recurrent prostate cancer in a high number of patients [61]. In line with these promising results, the impact of using ^68^Ga-PSMA-PET/CT in patients with recurrent prostate cancer is large, altering the therapeutic management in approximately half of all patients. Specifically, the use of dose escalation to boost the target volume and the proportion of focal salvage treatments seems to increase, while systemic treatment decreases [62].

#### 5.1.3. Biopsies

In the primary setting, it was shown that MRI-targeted biopsies, as opposed to transrectal ultrasonography (TRUS)-guided biopsies, decrease the detection of insignificant disease, while the yield of clinically relevant cancers increases [63]. A study in which patients subsequently underwent mp-MRI, TRUS-biopsies, and transperineal template prostate mapping (TPM) biopsies (sampling the whole gland every 5 mm) calculated that up to 18% more cases of clinically significant cancer might be detected if TRUS-biopsies were guided by MRI findings [50]. Adding mp-MRI information to subsequent TPM biopsies seems to achieve the highest diagnostic accuracy, with a sensitivity and specificity of 97% and 61%, respectively, a positive predictive value of 83%, and a negative predictive value of 91% [64]. Different approaches to achieve biopsy under MRI-guidance (i.e., in-bore, MRI/TRUS fusion, or cognitive registration) yield similar detection rates of clinically significant prostate cancer [65]. Interestingly, the definition of clinically significant cancer differs between studies, ranging from Gleason score 6 and cancer core length >3 mm to Gleason score ≥ 4 + 3.

In the radiorecurrent setting, prostate biopsy evaluation is hampered by radiation effects, which sometimes mimic higher-grade disease. Approximately 30% of indeterminate biopsies seem to resolve into negative disease status. On the other hand, local failure can also be interpreted as radiation effect, and indeterminate biopsies should, therefore, not be considered negative. Furthermore, delayed tumor regression may cause false positives. Biopsies should, therefore, not be taken before 24 months of follow-up [66]. Even after two years, routine post-radiotherapy biopsies are of limited added value to regular PSA testing, and should only be considered in case of biochemical failure [67]. According to the European Association of Urology (EAU) guidelines, biopsy after radiotherapy is only indicated if local recurrence affects treatment decisions [1].

In case of localized recurrence, one could argue that biopsies might aid in the selection of patients for focal salvage treatment. A study comparing cognitive targeted biopsies with TPM biopsies showed that targeted biopsies had similar or at most 10% less detection rate, depending on the definition of clinically significant cancer. Targeted biopsies were efficient, requiring fewer biopsies compared to TPM biopsies for detection of clinically significant disease [68]. However, clinical significance was determined based on either maximum cancer core length or Gleason score. Since the effect of altered architecture from previous radiotherapy on the Gleason score is poorly understood, it does not seem appropriate for grading radiorecurrent lesions [69,70,71]. Validation studies on the use of the Gleason scoring system in the radiorecurrent setting are lacking in the current available literature. Furthermore, there seems to be no consensus on the Gleason score definition for clinically significant disease. Histological confirmation of recurrence is, therefore, limited (i.e., adenocarcinoma yes/no) and does not provide any information on the clinical significance (tumor aggressiveness) of the recurrent lesion.

With advancements in imaging modalities as outlined above, and the burden of invasive biopsy procedures on patients, it is questionable whether these biopsies are mandatory for adequate disease assessment. There is no literature describing the accuracy of combined mp-MRI and PET-CT with pathology verification in the radiorecurrent setting. Currently, we are investigating a cohort of patients with a positive recurrent lesion on ^68^Ga-PSMA-PET/CT and at least one mp-MRI sequence, who underwent subsequent MRI-targeted biopsies, to determine the added value of histologic verification for adequate disease assessment.

### 5.2. Current Focal Salvage Series

Today, focal salvage treatment of radiorecurrent prostate cancer is performed with a variety of techniques: focal cryotherapy [72,73,74], focal HIFU [75], focal brachytherapy (both low-dose-rate [76,77] and high-dose-rate [78,79,80]), and, in smaller series, stereotactic body radiation therapy (SBRT) [81,82]. The extend of ablation differs per ablation method and between series, ranging from ultrafocal to hemi-ablation and subtotal ablation. Focal cryotherapy usually entails hemi-ablation by achieving a lethal freezing temperature of −40 °C in the prostate lobe containing the cancer. Focal HIFU can be hemi-ablation or quadrant ablation (one half of a lobe), using focused ultrasonic waves for tissue destruction by means of thermal, mechanical, and cavitation effects. With brachytherapy, ultrafocal ablation can be achieved by administering radiation to a small target volume, using the steep dose fall-off with distance from the radiation source. Iodine-125 seeds are used for low-dose-rate brachytherapy, delivering a prescribed dose of 144–145 Gy. High-dose-rate brachytherapy delivers radiation from an iridium-192 source through temporarily implanted catheters, which allow for dose painting by varying the dwell positions and times of the radiation source. High-dose-rate schedules vary from 18–19 Gy in a single dose to 27 Gy divided over two implants. CyberKnife-based SBRT is performed with dose schedules between 30–35 Gy in five fractions. While this technique offers a high degree of conformity, it is also likely to increase the integral dose to the surrounding healthy tissues. Furthermore, without real-time MRI guidance, planning target volume (PTV) margins for correction of intrafraction motion remain necessary to avoid geographical miss. Different focal ablation methods have varying limitations with respect to tumor recurrence location; HIFU is less suited for treating anterior-located lesions due to insufficient length of most devices, while cryotherapy can be less effective in the apical and peri-urethral region due to organ-protective warming tools. With brachytherapy, it is usually possible to cover all sides of the prostate [83,84].

Studies that report five-year bFFS seem to reach an approximate 50% rate [85], which is comparable to whole-gland salvage series. Only one study presented a direct comparison between focal and whole-gland using cryotherapy: five-year bFFS rates were 54 and 86%, respectively [72]. However, differences in patient characteristics and primary radiation schedules make it hard to interpret these results. Though most literature comes from relatively recent studies, patient selection methods are often already outdated. Exclusion of metastatic disease was often performed with either CT or MRI for nodal assessment, bone scintigraphy for bony disease, and, in some series, PET/CT in a small number of patients. A modern multimodal radiologic approach with mp-MRI and ^68^Ga-PSMA-PET/CT outperforms the other modalities in selecting patients with true localized, non-metastatic recurrence [44,86]. In the future, better patient selection could, therefore, improve oncologic outcomes of focal salvage series even further. Follow-up times are still too short to assess the impact of focal salvage treatment in terms of overall survival. However, the main impact lies in delaying the need for palliative hormonal treatment, while providing a chance of cure through local control.

With this in mind, it is important to consider treatment-related side-effects of focal salvage treatments. Although toxicity might be underreported in many current series due to the retrospective nature of data collection, the general trend seems favorable. Severe GU and GI toxicity seem limited to a maximum of 5–10%. Potency preservation (measured with the international index of erectile function (IIEF) or common terminology criteria for adverse events (CTCAE)) is observed in the majority of patients in many of the series. Treatment effects on patient-reported quality of life was only reported in focal salvage brachytherapy series, revealing no significant changes in most domains, except an increase in urinary symptoms after focal low-dose-rate brachytherapy [77].

Table 1 provides an overview of functional and oncologic outcomes of the different focal salvage treatment modalities.

**Figure 2:**
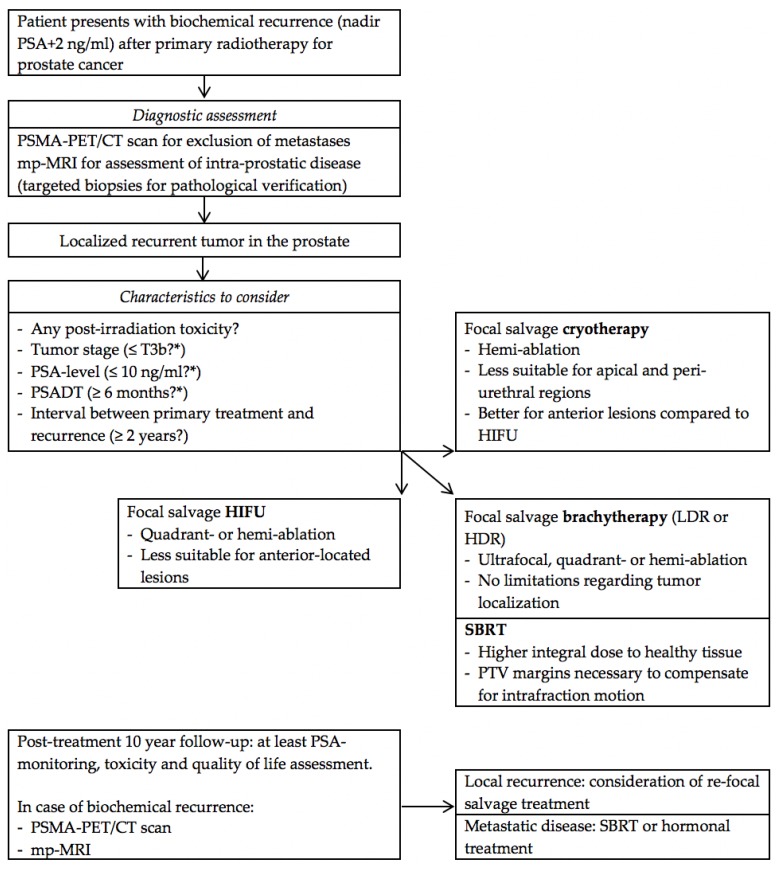
Flow chart for decision-making before and after focal salvage treatment of localized radiorecurrent prostate cancer. *Abbreviations*: PSMA: prostate-specific membrane antigen, mp-MRI: multiparametric magnetic resonance imaging, PSADT: PSA doubling time, HIFU: high-intensity focused ultrasound, LDR: low-dose-rate, HDR: high-dose-rate, SBRT: stereotactic body radiation therapy. * As proposed by Delphi consensus study among 18 experts in the field of salvage brachytherapy for radiorecurrent prostate cancer (conducted by UroGEC group of Groupe Européen de Curiethérapie/European Society for Radiotherapy and Oncology (GEC-ESTRO)) [87].

To determine which patients benefit the most from focal salvage treatment, it is also important to consider other patient and tumor characteristics. In the abovementioned studies, patients with stage T1–T3b recurrent tumors, total Gleason score ≤6–10, and PSA levels between 0.01 and ≥20 ng/mL were treated. This indicates that a wide range of patients, classified from (very) low-risk to high-risk disease, were included. Most studies did not report on the pre-treatment PSA doubling time (PSADT). In a Delphi consensus study among 18 experts in the field of salvage brachytherapy for radiorecurrent prostate cancer, 88% of participants indicated that stage T3b should be the maximum tumor classification to be eligible for salvage treatment. A total of 94% agreed that the Gleason score should not be used as a criterion (with over half of participants stating that the Gleason score cannot be determined in case of relapse after primary radiotherapy). In terms of PSA kinetics, a maximum PSA level of 10 ng/mL and minimum PSADT of six months was preferred by most participants [87]. A prediction study on factors associated with failure after focal salvage HIFU revealed that the length of the interval between primary treatment and radiologic recurrence, prostatic volume, T-stage, PSA level, PSADT, and primary tumor Gleason score are potential predictors of failure [88]. More research is warranted to better understand which combination of patient and tumor characteristics is best served by (which) focal salvage treatment. The decision-making process before and after focal salvage treatment is displayed in a flow chart in Figure 2.

### 5.3. Future Prospects Regarding MRI-Guided Radiotherapy

It is clear that accurate targeted ablation requires precise localization of the recurrent prostatic lesion. Over the years, the use of (mp-)MRI for treatment planning substantially increased. The superior resolution of soft tissue enables more accurate delineation of the tumor volume and organs at risk [89]. New developments such as ultra-high-field MRI with 7-T systems have the potential to enhance the spatial resolution even further [90]. Although it seems that 7-T T2- and diffusion-weighted imaging deliver clinically adequate anatomical images within acceptable acquisition times, there are still several technical challenges to overcome before a 7-T mp-MRI protocol for the prostate can be achieved [91].

Imaging developments are not only used for the treatment planning phase, but are also increasingly incorporated into the treatment itself. Currently, MRI guidance during treatment can be achieved using image registration of pre-operative MR images (1.5 T or 3 T) with intra-operative TRUS images (MRI/TRUS fusion). With this technique, software is used to register the pre-operatively delineated tumor location to real-time prostate images. Image registration may be either rigid (overlay of images without adjustment for possible prostate deformation during treatment) or non-rigid (using algorithms that compensate for deformation). Some factors that contribute to prostate deformation are unavoidable, such as swelling of the prostate due to catheter insertion during a brachytherapy implant procedure. Prostate motion can also be caused by surrounding organ movement, such as rectal distension due to flatulence or introduction of an ultrasound probe. Evidently, non-rigid registration is challenging; a variety of registration methods using different algorithms were presented in the search for the most optimal solution [92].

The next step in the development of MRI-guided intervention is the incorporation of live MR images into the treatment workflow, thereby achieving direct treatment guidance and avoiding any registration errors. Although early experiences with real-time MRI-guided brachytherapy date back to 1997, this approach is not yet widely adopted due to logistical issues such as resource demand and procedural time prolongation [93]. One of the obvious challenges of in-bore intervention is the limited workspace. Open MRI units that provide access to the patient while imaging are available, but these deliver low image quality and need increased scanning time due to the inherently lower signal-to-noise ratio.

**Figure 3:**
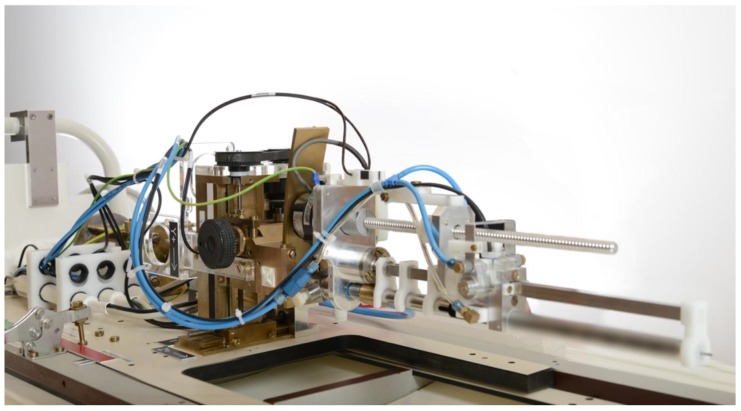
Magnetic resonance imaging (MRI)-compatible robotic implantation device for prostate brachytherapy. A cylindrical weight that is pneumatically driven hits the needle holder to tap a brachytherapy needle into the prostate. When placed between the patient’s legs inside an MRI scanner, the needle can be tracked using live images.

To overcome these shortcomings, a robotic MRI-compatible implantation device for prostate brachytherapy was developed at our institution (see Figure 3). The robot system fits in a 1.5-T MRI scanner and can be placed between the patient’s legs. In 2010, the first clinical proof of principal study was performed with the University Medical Center Utrecht (UMCU) robot, successfully implanting gold fiducial markers into the prostate for external beam radiation [94]. It was shown that the in vivo use of the robot was feasible. After this first clinical test, the UMCU robot was further developed and optimized for the application of brachytherapy implant procedures. We are currently working on a study investigating the in vivo technical feasibility of robotic insertion of a brachytherapy needle into the prostate. It is expected that this study will be a step forward in the development of MRI-guided focal salvage brachytherapy with a robotic device. In the future, a full MRI-guided robotic implantation procedure may allow for a reduction of needles needed for the implant [95], with expected lower toxicity rates and a reduction of time necessary for the procedure.

Regarding external beam radiotherapy, MRI-guided radiotherapy systems such as the MR-Linac will provide another way of accomplishing live MRI-guided intervention. Using online fast MR-sequences for auto-contouring and auto-planning, a full MRI-based online adaptive workflow can be achieved [96]. Changes in anatomy can be accounted for with inter-beam replanning. This will further reduce the target volume margins needed, reducing normal tissue radiation exposure and thereby decreasing the risk of toxicity. This enables safe dose escalation, potentially in the form of delivering a single ablative dose, which would be of benefit to both patient comfort and hospital logistics. It should, however, be noted that external beam radiotherapy is inherently less conformal than brachytherapy, and it remains to be seen whether this treatment modality will be suitable for focal treatment in the recurrent prostate cancer setting.

## 6. Conclusions

Localized radiorecurrent prostate cancer seems susceptible to focal salvage treatment. Treating the tumor while sparing the surrounding healthy tissue leads to a reduction of treatment-related side-effects, where whole-gland salvage treatments or palliative ADT are often less well tolerated. Focal salvage therapy thereby provides an intermediate step between primary curative treatment and (if necessary) palliative hormonal treatment. Diagnostic innovations led to more adequate patient selection in terms of exclusion of metastatic disease and accurate tumor targeting. This is a constantly developing field, as new diagnostic techniques are warranted to provide greater insight into prostate tumor profiling. With MRI guidance, focal treatment becomes more and more precise, especially with emerging technologies enabling live and online adaptive MRI-guided radiotherapy.

## Figures and Tables

**Table 1:** Summary of studies on functional and oncologic outcomes of different focal salvage treatment modalities for localized radiorecurrent prostate cancer.

Focal Salvage Treatment	Study	Ablation Extent	Patients	Median Follow-up	bFFS	GU/GI Toxicity	QoL
Brachytherapy							
LDR	Kunogi et al. [76]	Ultrafocal (145 Gy)	12	56 months	78% at 4 years	No grade 3	NA
	Peters et al. [77]	Ultrafocal (144 Gy)	20	36 months	60% at 3 years	5% grade 3 GU	Increase in urinary symptoms
HDR	Zamboglou et al. [78]	Ultrafocal (18 Gy)	2	6 months	100% at 6 months	No grade 3	NA
	Maenhout et al. [79]	Ultrafocal (19 Gy)	17	10 months	92% at 1 year	6% grade 3 GU	NA
	Murgic et al. [80]	Quadrant (27 Gy in 2 fractions)	15	36 months	61% at 3 years	7% grade 3 GU	No significant change
Cryotherapy	de Castro Abreu et al. [72]	Hemi	25	31 months	54% at 5 years	No incontinence, no fistula	NA
	Kongnyuy et al. [73]	Hemi	65	27 months	48% at 3 years	6% incontinence	NA
	Li et al. [74]	NA	91	15 months	47% at 5 years	6% incontinence, 7% retention, 3% fistula	NA
HIFU	Kanthabalan et al. [75]	Ultrafocal (11%), quadrant (55%), hemi (34%)	150	35 months	48% at 3 years	8% bladder neck stricture, 2% fistula	NA
SBRT	Jereczek-Fossa et al. [81]	Ultrafocal (30 Gy in 5 fractions)	15	10 months	22% at 2.5 years	7% grade 3 GU	NA
	Mbeutcha et al. [82]	Ultrafocal (35 Gy in 5 fractions)	18	15 months	56% at 1 year	No grade 3	NA

Abbreviations: bFFS: biochemical failure-free survival, GU: genitourinary, GI: gastrointestinal, QoL: quality of life, LDR: low-dose-rate, HDR: high-dose-rate, HIFU: high intensity focused ultrasound, NA: not available, SBRT: stereotactic body radiation therapy.
